# Exosomal miR-17 Drives Thyroid Cancer Lung Metastasis via NF-**κ**B Activation

**DOI:** 10.32604/or.2025.067182

**Published:** 2025-11-27

**Authors:** Yan Gui, Wen Pan, Ziyi Dong, Dongzhi Hu, Yaoyang Guo, Xinyi Wen, Haiyang Zhang, Zhansheng Jiang, Xiangqian Zheng, Ming Gao, Junyi Wang

**Affiliations:** 1 The First Hospital of Lanzhou University, The First School of Clinical Medicine, Lanzhou University, Department of Otorhinolaryngology Head and Neck Surgery, Tianjin Medical University Cancer Institute and Hospital, Lanzhou, 730030, China; 2 Tianjin Institute of Coloproctology, Tianjin Union Medical Center, The First Affiliated Hospital of Nankai University, Tianjin, 300121, China

**Keywords:** Thyroid cancer, exosomes, microRNA-17, inflammatory environment, tumor metastasis

## Abstract

**Objectives:**

Metastatic spread to the lung is one of the leading causes of fatal outcomes in thyroid cancer, but the underlying molecular mechanisms remain unclear. To investigate how exosomal microRNA-17-5p (miR-17-5p) promotes lung metastasis in thyroid cancer within the framework of the “seed and soil” hypothesis.

**Methods:**

Serum exosomes from thyroid cancer lung metastasis patients and controls were analyzed for miR-17, which was elevated in metastatic cases. miR-17 was transfected into embryonic lung fibroblasts (MRC-5), and their supernatants were co-cultured with thyroid cancer cells (Cal62). Cell proliferation and migration were evaluated using colony formation, Ki67 staining, and Transwell assays. Interleukin-6 (IL-6)/interleukin-8 (IL-8) levels and nuclear factor kappa-B (NF-κB)/nuclear factor kappa-B repressing factor (NKRF) expression were analysed by enzyme-linked immunosorbent assays (ELISA) and western blot. *In vivo* models verified the metastatic-promoting effect of miR-17.

**Results:**

miR-17-5p was significantly enriched in serum exosomes of metastatic patients. In MRC-5 cells, it suppressed NKRF, NF-κB signaling, and increased secretion of IL-6 and IL-8, enhancing Cal62 proliferation and migration. Animal experiments confirmed its role in promoting tumor growth and lung metastasis.

**Conclusions:**

Exosomal miR-17-5p remodels the pulmonary microenvironment into a pro-inflammatory niche, facilitating thyroid cancer colonization and offering a potential therapeutic target.

## 1 Introduction

Thyroid cancer has been reported to show a steadily increasing incidence worldwide in recent years, raising significant public health concerns [1]. Among all subtypes, differentiated thyroid cancer (DTC) has been identified to account for over 95% of cases. The majority of differentiated thyroid cancers exhibit slow clinical progression and favorable outcomes, with 10-year survival rates reaching ~96% in the United States [2] and 70%–86% in China [3]. However, this prognosis is markedly reduced once distant metastasis occurs [2]. In differentiated thyroid cancer, the lung is identified as the predominant site of distant metastasis [4,5], and pulmonary metastasis has been acknowledged as a key driver of thyroid cancer-related mortality [6]. Therefore, the prompt detection and definitive diagnosis of lung metastasis in DTC are considered critical for improving patient outcomes and remain a primary objective in treating advanced thyroid cancer.

The “seed and soil” hypothesis proposes that tumor metastasis is not a stochastic event but depends on the compatibility between circulating malignant cells (“seeds”) together with the organ-specific microenvironment (“soil”) [7,8]. Recent studies have extended this concept by showing that primary tumors can actively modify distant tissues [9,10]. This mechanism results in the establishment of a pre-metastatic niche, creating a supportive microenvironment that facilitates the survival and growth of disseminated tumor cells prior to the development of detectable metastases [11].

As small extracellular vesicles derived from cells, exosomes play a pivotal role in preparing distant organs for tumor metastasis [12,13]. Tumor-derived exosomes are enriched with microRNAs (miRNAs), messenger RNAs (mRNAs), and signaling proteins, and can travel through the circulation to distant organs, where they modulate local signaling pathways and reprogram the microenvironment [14]. In pancreatic, breast, and melanoma models, exosomes have been shown to facilitate metastasis by establishing supportive niches in target organs [15–17]. However, the contribution of exosomes to remodeling the pulmonary niche in thyroid cancer metastasis remains largely unclear.

miRNAs, a class of short non-coding RNAs with an average length of ~22 nucleotides, function as critical regulators of gene expression through post-transcriptional mechanisms. It has been demonstrated that miRNAs encapsulated in exosomes can be internalized by recipient cells, where they bind to target mRNAs and inhibit gene expression. Through this mechanism, miRNAs have been implicated in tumor initiation, progression, metastasis, and in remodeling the tumor microenvironment (TME). They are intimately linked to various aspects of cancer development and dissemination [18,19].

Although thyroid cancer generally exhibits a favorable prognosis [2], a subset of patients develops distant metastases, particularly to the lungs, which are associated with poor clinical outcomes and limited treatment options [4]. Growing evidence has indicated that extracellular vesicles (EVs), including exosomes, play a key role in mediating intercellular communication within the TME. These vesicles are capable of transferring bioactive molecules such as miRNAs, which regulate critical processes including inflammation, immune modulation, and tumor progression [14]. Among these, microRNA-17 (miR-17) has been reported to modulate inflammatory pathways, including nuclear factor kappa-B (NF-κB) signaling, in various cancer types [18]. Despite these advances, the precise mechanisms by which exosomal miRNAs drive pre-metastatic niche formation in thyroid cancer remain largely unclear. Therefore, this study aimed to determine whether exosomal miR-17 contributes to thyroid cancer metastasis by altering the pulmonary microenvironment.

## 2 Materials and Methods

### 2.1 Human Samples

Preoperative serum was obtained from patients diagnosed with differentiated thyroid cancer presenting with lung metastases or cervical lymph node metastases, as well as from age-and sex-matched healthy volunteers. Samples were from the Airport Hospital of Tianjin Medical University Cancer Hospital and its affiliated centers. In total, 50 serum specimens from patients affected by metastatic lesions in cervical lymph nodes and their respective controls were analyzed. Written consent was obtained from every participant in accordance with ethical requirements. The study protocol was reviewed and approved by the Ethics Committee of Tianjin Medical University Cancer Institute and Hospital (Approval number: EK20240371). All procedures adhered to applicable ethical standards and regulatory requirements.

### 2.2 Animals

Female nude mice (Spacepharma Beijing Biotechnology Co., Ltd., Beijing, China) (BALB/c-nu, 4–6 weeks, 18–20 g, n = 15) were housed in an SPF-grade animal facility at the Cancer Hospital of Tianjin Medical University. Animal experiments were performed following protocols approved by the ethics committee approved by the Institutional Animal Care and Research Advisory Committee of the Cancer Institute and Hospital of Tianjin Medical University (Approval number: NSFC-AE-2024148). The study followed the ARRIVE reporting guidelines (https://arriveguidelines.org).

### 2.3 Cell Lines and Cell Culture

Human thyroid cancer Cal62 cells were obtained from the Cell Bank of the Chinese Academy of Sciences (Shanghai, China), and were cultured in Dulbecco’s Modified Eagle’s Medium (DMEM) (Gibco, 11-879-020, Grand Island, NY, USA) supplemented with 10% bovine serum (FBS) (Gibco, U3196808P). Human embryonic lung cells MRC-5 (Zhongqiao Xinzhou Biotechnology Co., Ltd., ZM0006, Shanghai, China) were cultured in MEM medium supplemented (Gibco, 11095080) with 10% bovine serum (Gibco, C0235), 1% L-alanyl-glutamine (Beyotime, C0211-100ml, Shanghai, China), 1% penicillin-streptomycin (Gibco, 1514012), and 1% sodium pyruvate (Beyotime, Y025073, Shanghai, China) were added to the medium. All cells were cultured at 37°C with 5% carbon dioxide, validated as mycoplasma-free and authenticated by STR analysis prior to use.

### 2.4 Isolation of Exosomes

Following established protocols, exosomes in MEM culture medium and serum were isolated according to the method described previously [20]. Initially, cells and cellular debris were pelleted by centrifugation at 3000× *g* for 15 min, followed by removal of large vesicles through centrifugation at 10,000× *g* for 15 min. Finally, the exosomes were ultracentrifuged at 110,000× *g* for 90 min and the exosomes were collected as pellets after ultracentrifugation and resuspended in 1× PBS (pH 7.4, Beyotime, C0221A, Shanghai, China). All steps were performed at 4°C. Exosomes were stored at −80°C after extraction.

### 2.5 Nanoparticle Tracking Analysis (NTA)

Particle size distribution and concentration were measured using the NanoSight NS 300 system (NanoSight Technology, Malvern, UK). Exosomes were suspended in 1× PBS (pH 7.4; Beyotime, C0221A) at 5 μg/mL, diluted 100–300-fold to obtain 20–100 particles/frame, and analyzed at room temperature using a 488 nm laser with triplicate detection on an sCMOS camera (Oxford Instruments Andor Ltd., Belfast, UK). The data was then analysed with NTA (version 2.3, Malvern Panalytical, Malvern, UK).

### 2.6 Transmission Electron Microscope (TEM) Assay

Exosomes were washed 3 times (10 min each) in 1× PBS (pH 7.4, Beyotime, C0221A) buffer and then fixed 60 min at room temperature by using 1% osmium tetroxide (TCI Shanghai, O0308, Shanghai, China). The exosome pellets were then embedded in 10% gelatin (Beyotime, Y263031, Shanghai, China), fixed at 4°C in glutaraldehyde (Beyotime, Y241202, Shanghai, China), and cut into pieces (less than 1 mm). The samples were then dehydrated in increasing concentrations of alcohol (30%, 50%, 70%, 90%, 95% and 100% × 3, each step for 10 min). Samples underwent infiltration with Quetol-812 epoxy resin in gradually increasing concentrations (HEAD Biotechnology Co., Ltd., SPI-02660, Beijing, China) and propylene oxide (J&K scientific, E0016, Shanghai, China) mixture (25%, 50%, 75% and 100%, 3 h, each step). Embedding was performed using freshly prepared Quetol-812 epoxy resin (HEAD Biotechnology Co., Ltd., SPI-02660) and polymerised at 35°C (12 h), at 45°C (12 h), and at 60°C (24 h). Leica UC6 ultramicrotome was used to obtain ultrathin sections (~100 nm) (Leica Microsystems GmbH, Wetzlar, Germany), stained with 2% uranyl acetate (HEAD Biotechnology Co., Ltd., SPI-02624, Beijing, China) for 10 min, followed by lead citrate (prepared according to Reynolds’ method) (HEAD Biotechnology Co., Ltd., HD17810, Beijing, China) for 5 min. A FEI Tecnai T20 TEM (Field Electron and Ion Company, Hillsboro, OR, USA) was used to visualize the samples.

### 2.7 Western Blotting (WB) Analysis

Total protein was extracted from cultured MRC-5 cells and animal tissue samples. Cells were lysed using RIPA lysis buffer (Beyotime, P0013C, Shanghai, China) supplemented with protease inhibitor (Beyotime, P1005). Tissues were homogenized in pre-chilled RIPA lysis buffer, then centrifuged at 12,000× *g* for 15 min at 4°C, and the supernatant was collected. Protein concentration was determined using the BCA Protein Quantification Kit (Beyotime, P0012S). Equal amounts of protein (10 μg) were separated by 10% SDS-PAGE (Beyotime, P0562) and transferred to a PVDF membrane (Beyotime, FFP33) at 280 mA for 120 min. Membranes were blocked with 5% nonfat dry milk in TBST (Beyotime, ST671) for 1 h, followed by overnight incubation at 4°C with the following primary antibodies: anti-NKRF (1:1000, Santa Cruz, sc-365568), anti-NF-κB (1:1000, Santa Cruz, sc-8008), anti-TSG101 (1:5000, Proteintech, 67381-1-Ig), anti-Alix (1:1000, Proteintech, 67715-1-Ig), anti-CD9 (1:1000, CST, 13403S), anti-GAPDH (1:2000, Bioss, bs-0755R). After washing the membrane, add HRP-labeled secondary antibody and incubate for 1 h, then wash the membrane again. Protein bands were visualized using ECL reagent (Beyotime, C0221A) and detected on a gel imaging system (Thermo Fisher Scientific, iBright FL1500, Carlsbad, USA). GAPDH served as an internal control.

### 2.8 Dual-Luciferase Reporter Assay

MRC-5 cells at 50%–80% confluence in 12-well plates were co-transfected with wild-type (NKRF WT) or mutant (NKRF Mut) luciferase reporter plasmids and miR-17-5p mimics or negative control (NC) mimics (GeneChem, Shanghai, China) using Lipofectamine 3000 (Thermo Fisher Scientific, L3000015). Luciferase activity was assessed 48 h post-transfection with a dual-luciferase reporter assay system (Promega, E1910) and a luminometer (Thermo Fisher Scientific, Luminoskan Ascent). NC mimics with NKRF WT or NKRF Mut served as negative controls, NKRF WT plus miR-17-5p mimics as a positive control, and Renilla luciferase plasmid was co-transfected for normalization.

### 2.9 PKH26 Staining for Exosomes

PKH26 Fluorescent Cell Linker Kit was used to stain exosomes (Sigma, PKH26PCL, Saint Louis, MO, USA). A working dye solution was prepared by mixing 200 μL of Diluent C with 0.8 μL of PKH26 dye. Exosomes were measured on a NanoDrop 2000 spectrophotometer (Thermo, Waltham, MA, USA), then resuspended in 200 μL of Diluent C and incubated with the dye solution for 3 min. The labeling reaction was terminated by adding 400 μL of fetal bovine serum (Gibco, C0235) for 1 min. After being washed twice with PBS (Beyotime, C0221A), and co-incubated with recipient cells in 24-well plates for 4–6 h. The nuclei of the cells were then stained, and finally the cells were examined for exosome uptake by confocal microscopy (Nikon, C2 Plus, Tokyo, Japan).

### 2.10 Immunofluorescence

Approximately 5 × 10^4^ MRC-5 and Cal62 cells were seeded per well on twenty-four well chamber slides and cultured until reaching appropriate confluency. Cells underwent fixation with 4% paraformaldehyde (Beyotime, P0099) and subsequent permeabilization in 0.2% Triton X-100 (Beyotime, ST1723) for 10 min. Non-specific binding was blocked using 1% BSA and 10% normal goat serum in PBS at room temperature for 1 h. The cells were then treated with anti-Ki67 (1:1000; Abcam, ab15580, Cambridge, UK) antibody. In addition, antibody staining was performed with rabbit-derived fluorescent secondary (1:200, Zhongshanjinqiao, ZF-0516) antibody (594 nm). All cells were stained for nuclei with 1 μg/m 4^′^,6-diamidino-2-phenylindole dye (358 nm) (cidabio, Bio-CD2477, Guangzhou, China). Staining was completed and blocked with an anti-fluorescent attenuator (Beyotime, P0131, Shanghai, China). Confocal microscopy was used with a Zeiss confocal microscope (Zeiss, LSM 880, Jena, Germany).

### 2.11 RNA Isolation and Quantitative (RT-PCR)

TRIzol Reagent was employed to isolate total RNA from MRC-5 and Cal62 cells (Thermo Fisher Scientific, 15596026CN, Carlsbad, CA, USA) according to the manufacturer’s instructions. cDNA was synthesized from 1 μg of total RNA by reverse transcription, and 2 μL of cDNA was used for miRNA quantification on a real-time qPCR system (Eppendorf, AG 22331, Hamburg, Germany). Relative gene expression levels were calculated using the 2^−ΔΔCT^ method, where ΔΔCT = (CT_target − CT_reference)_treatment − (CT_target − CT_reference)_control. Primers for human miR-17-5p were: forward, CAAAGTGCTTACAGTGCAGGTAG; reverse, ATCCAGTGCAGGGTCCGAGG.

Cells transfected with overexpression vectors (OE) or shRNA plasmids (sh) targeting miR-17-5p were used as positive treatment groups, while cells transfected with non-targeting overexpression (OE NC) or shRNA control vectors (sh NC) served as negative controls.

### 2.12 Cell Proliferation Assays

To evaluate cell proliferation, we performed a colony formation assay. Briefly, we plated 500 Cal62 cells in each well of a six-well plate. After an initial week of culture, the medium was refreshed every other day for another week. After a total of two weeks, the medium was removed, and the cell colonies were fixed with methanol for 15 min (Beyotime, Y037896, Shanghai, China), followed by incubation in 0.1% crystal violet (Beyotime, C0121, Shanghai, China). Observe the cell proliferation.

Cells were treated with 50 μM EdU (Ruibo Bio, Guangzhou, China) for 12 h, then fixed with 4% paraformaldehyde (Beyotime, P0099) for 30 min permeabilized in 1× PBS (Beyotime, C0221A) containing 0.2% Triton X-100 (Beyotime, ST1723) for 10 min, and stained according to the protocol (Ruibo Bio, C10310). Images were captured using a Zeiss confocal microscope.

### 2.13 Cell Migration Assays (Transwell)

The Cal 62 cell migration assay was performed using Transwell Boyden chambers (6.5 mm, polycarbonate membrane, Corning, New York, USA). Cells were seeded at 1 × 10^5^ cells/200 μL in serum-free DMEM (Gibco, 11-879-020) into the upper chamber, while the lower chamber was filled with 500 μL DMEM containing 20% FBS (Gibco, C0235). After incubating the culture plates at 37°C for 6–8 h, cells migrating to the lower surface of the membrane were fixed with ice-cold methanol (Biosharp, Y037896) for 15 min and stained with 0.1% crystal violet (Biosharp, C0121) for 15 min. Migrating cells were imaged using a Nikon Eclipse Ts2 inverted microscope (Nikon, Japan).

### 2.14 Enzyme-Linked Immunosorbent Assays (ELISA)

We used an ELISA method to determine the concentrations of interleukin-6 (IL-6) and interleukin-8 (IL-8) in culture supernatants. The experiment was performed using commercial kits from MEIBIAO (Taizhou, China; Cat. Nos. MB-0049B, MB-0088B) according to the manufacturer’s instructions. The procedure involved incubating 100 μL of samples or standards at 37°C for 60 min. After washing, incubating with 100 μL of HRP-conjugated detection antibody for 30–60 min. After a second wash, adding 100 μL of TMB substrate and incubating in the dark for 10–15 min, and finally adding 50 μL of 2 M H_2_SO_4_ stop solution. We calculated the final concentrations by measuring the Optical Density (OD) at 450 nm and referring to a four-parameter logistic (4-PL) standard curve.

### 2.15 Co-Immunoprecipitation (Co-IP)

MRC-5 cells were immunoprecipitated using anti-NKRF antibody (sc-365568, Santa Cruz) 48 or 72 h after infection. Cells were digested with 0.25% trypsin (Gibco, 25200072, New York, USA) and then lysed in lysis buffer containing 150 mM KCl (Beyotime, Y000949, Shanghai, China), 25 mM Tris-HCl, pH 7.4 (Beyotime, ST774, Shanghai, China), 5 mM EDTA (Beyotime, Y257063, Shanghai, China), 0.5% Triton X-100 (Beyotime, ST1723), 5 mM dithiothreitol (DTT), PMSF (Thermo Fisher Scientific, 36978, Carlsbad, CA, USA), and cocktail, four times for 10 min. The lysates were centrifuged at 12,000 rpm (Eppendorf 5424, Eppendorf, Hamburg, Germany) for 10 min. The supernatants were incubated overnight at 4°C with an anti-NKRF antibody (sc-365568, Santa Cruz) to form immune complexes. Subsequently, beads (Santa Cruz) were added, and the mixture was further incubated at room temperature for 2–4 h to capture the complexes. Following five washes with lysis buffer (Invitrogen, FNN0021, Shanghai, China), the final immunoprecipitates were analyzed by WB. For Co-IP experiments, input lysates were used as positive controls, while normal IgG served as a negative control to confirm antibody specificity.

### 2.16 Transfection

For miRNA transfection experiments, negative control (NC) mimics, human miR-17-5p mimics, and human miR-17-5p inhibitors were obtained from (RiboBio, Guangzhou, China). MRC-5 cells transfected with NC mimics served as the negative control group, while cells transfected with miR-17-5p mimics or inhibitors were used as positive treatment groups to assess the gain-and loss-of-function effects of miR-17-5p, respectively. Cells were transfected with Lipofectamine 2000 (Invitrogen, Carlsbad, CA, USA) following the manufacturer’s instructions and collected 48 h later for analyses. The miR-17-5p mimic, inhibitor, and corresponding negative controls used in this study were commercially synthesized products (RiboBio, Guangzhou, China; catalog numbers as listed below):

miR-17-5p mimic: miR1CM001

miR-17-5p inhibitor: miR2CM00

Negative control mimic (micrON mimic NC #22): miR1N0000001-1-5

Negative control inhibitor (micrOFF inhibitor NC #22): miR2N0000001-1-5

For experiments involving NF-κB pathway inhibition, cells were pretreated with 100 μM JSH-23 (Selleckchem, Houston, TX, USA) for 1 h prior to transfection.

### 2.17 Establishment of the Tumour Model in Nude Mice

All animal experiments were approved by the Ethics Committee of Tianjin Medical University Cancer Institute and Hospital (Approval No. NSFC-AE-2024148) and adhered to the ARRIVE Essential 10 guidelines. Female BALB/c-nu mice (Spacepharma, Beijing, China; 4–6 weeks, 18–20 g) were acclimatized for one week in an SPF facility with free access to nourishment before being randomized into groups (n = 5). Animals were monitored daily and euthanized by CO_2_ inhalation and cervical dislocation if predefined humane endpoints were reached (tumor ≥ 15 mm, >20% weight loss, or distress).

Xenograft model: Cal62 cells (5 × 10^6^ in 100 μL 1× PBS, pH 7.4) were injected subcutaneously into the groin. When tumors reached ~2–3 mm (day 7), mice received tail vein injections of exosomes (100 μL) or 1× PBS (pH 7.4, Beyotime, C0221A) every other day.

Lung metastasis model: Cal62 cells transfected with miR-17 (1 × 10^6^ in 100 μL 1× PBS, pH 7.4) were injected via the tail vein. Lung metastases were subsequently assessed after 4 weeks by *in vivo* imaging, micro-CT, and histology.

### 2.18 Next-Generation Sequencing (NGS)

Total RNA extraction from serum-derived exosomes was performed with the miRNeasy Micro Kit (QIAGEN, Hilden, Germany), followed by a quality and quantity assessment using a NanoDrop 2000 spectrophotometer and an Agilent 2100 Bioanalyzer. Small RNA libraries were generated with the NEBNext Small RNA Library Prep Kit (NEB, Ipswich, MA, USA) and subjected to sequencing on the Illumina HiSeq platform. The bioinformatics pipeline involved filtering the raw reads for quality, mapping the clean reads to the human reference genome (GRCh38), and annotating them against miRBase v22.

### 2.19 In Vivo Imaging and Micro-Computed Tomography (Micro-CT)

At week 4 after tail vein injection, *in vivo* imaging was performed under anesthesia using an IVIS Spectrum small animal imaging system (PerkinElmer, Waltham, MA, USA). Mice were imaged to detect lung metastases, and the images were analyzed using the system’s software (Living Image Software, PerkinElmer). At week 5, micro-CT scanning was performed using a Quantum GX micro-CT small animal scanner (PerkinElmer, Waltham, MA, USA) to evaluate the distribution and volume of lung lesions.

### 2.20 Hematoxylin and Eosin (H&E) Staining

For histological analysis, harvested lung tissues were first preserved in 4% paraformaldehyde (Beyotime, Shanghai, China), embedded in paraffin, and sectioned at 4 μm thickness using a Leica RM2235 microtome (Leica Microsystems, Wetzlar, Germany). Samples were incubated in hematoxylin for 5 min and eosin for 2 min (Sigma-Aldrich), then dehydrated through graded ethanol, cleared with xylene, and mounted using neutral resin, and visualized on an Olympus BX53 microscope (Olympus, Tokyo, Japan).

### 2.21 Data Analysis and Statistical Methods

All experiments were performed three times and all quantitative data are expressed as mean ± standard deviation (s.d.). Statistical tests were performed using the Student’s *t*-test in the GraphPad Prism system (GraphPad Prism 9.0, GraphPad Software, San Diego, CA, USA). No samples were excluded from the analysis. *p* < 0.05 is considered significant. ****p < 0.0001; ***p < 0.001; **p < 0.01; *p < 0.05; ns, not significant.

## 3 Results

### 3.1 Exosomes miRNA Is Associated with Lung Metastasis of Thyroid Cancer

miRNAs are known to regulate gene expression by targeting and inhibiting miRNAs in recipient cells, a process often mediated by exosomes. These miRNAs are involved in tumor initiation, progression, and metastasis, and are closely associated with cancer development [20–22]. However, their role in thyroid cancer metastasis remains insufficiently characterized.

**Figure 1:**
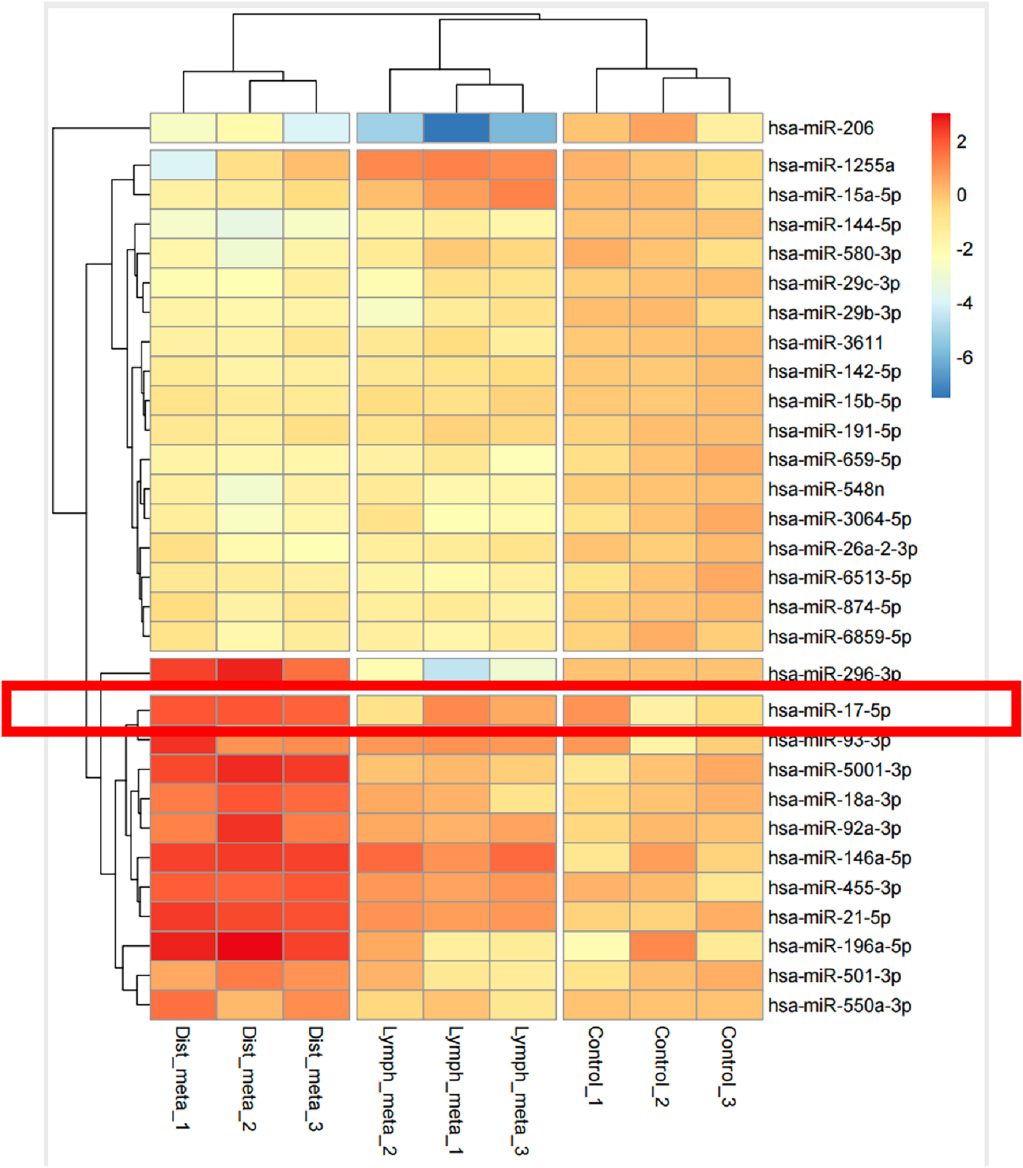
miRNAs carried in exosomes are related to the development of lung metastasis in thyroid cancer

In the present study, serum specimens were obtained from thyroid cancer patients with lung metastases. The (Next-Generation Sequencing) NGS analysis revealed significant alterations in miRNA levels in serum-derived exosomes from individuals with pulmonary metastases compared to those with lymph node metastases and non-metastatic disease (Fig. 1). Notably, miRNA levels were markedly elevated in patients with lung metastases relative to healthy controls. These results indicate a possible link between exosome-derived miRNAs and lung metastasis in thyroid cancer, offering a rationale for further studies.

### 3.2 Serum Exosomes Enhance the Ability of Thyroid Cancer Cells

**Figure 2:**
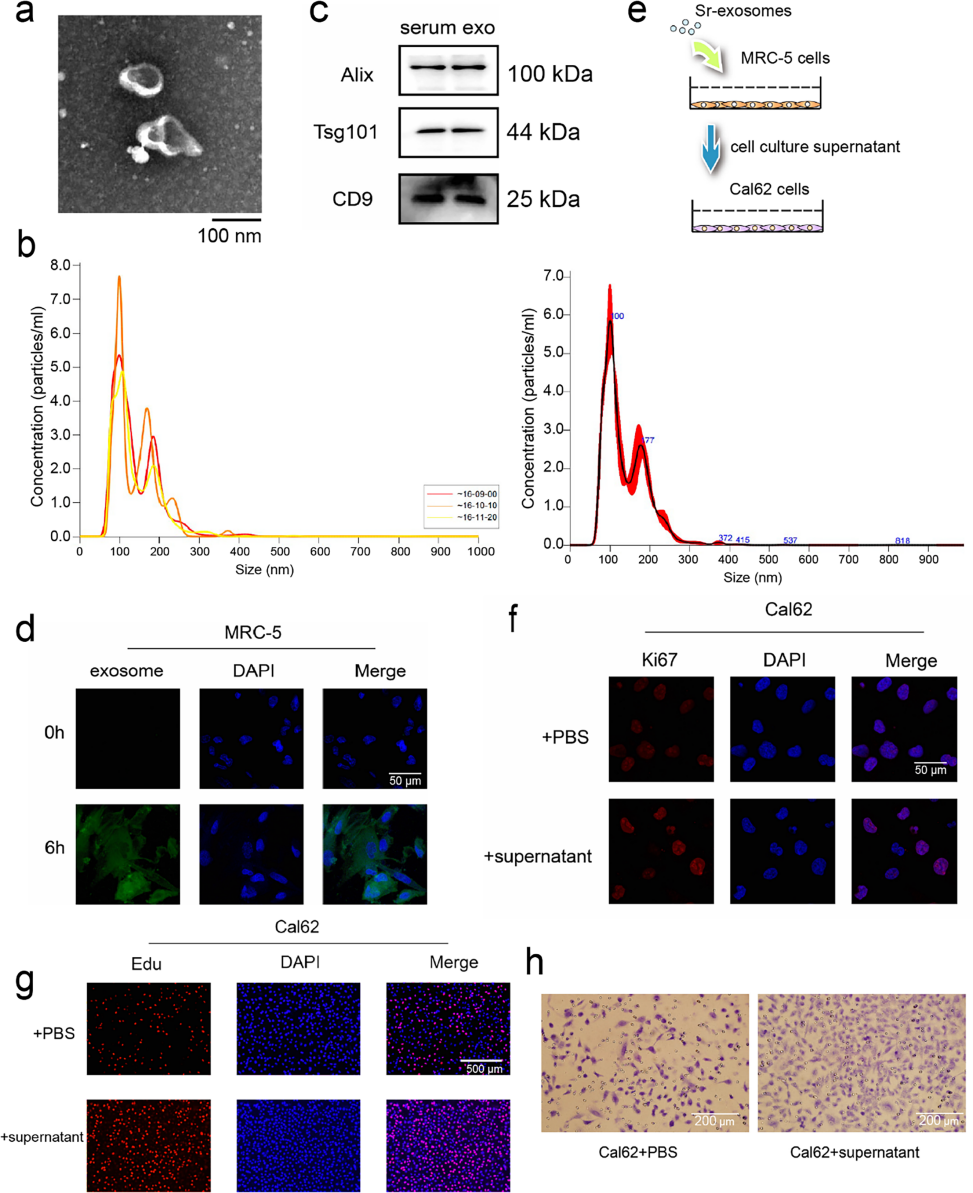
miRNAs carried in serum exosomes promote the ability of thyroid cancer cells. (**a**,**b**) Serum exosomes in individuals with thyroid cancer–associated lung metastases were identified by Transmission electron microscope (TEM) (**a**) and Nanoparticle tracking analysis (NTA) (**b**). (**c**) Western blot (WB) detection of exosomal markers in purified exosomal proteins. (**d**) Fusion of serum exosomes with embryonic lung cells in patients with thyroid cancer lung metastases was detected by exosome tracer assay. (**e**) Schematic diagram of exosome and cell co-incubation. (**f**,**g**) Immunofluorescence and Edu assay to evaluate the proliferative potential of thyroid cancer cells following co-culture (**h**) Cell migration assay to evaluate the migratory capacity of thyroid cancer cells following co-culture

Lung metastasis in thyroid cancer has been associated with miRNAs carried by serum-derived exosomes, which are known to influence tumor cell behavior, including proliferation and metastatic potential [19,23–25]. To investigate whether exosomes purified from patient serum samples with thyroid cancer lung metastases exert similar effects, exosomes were isolated from patient serum samples and their identity confirmed by NTA, TEM, and WB for canonical exosomal markers (Fig. 2a,c). NTA showed that the majority of particles were distributed between 60 and 200 nm, with a primary peak at approximately 100 nm and a secondary peak at 177 nm. The particle concentration at the primary peak reached up to ~6.5 × 10^7^ particles/mL (Fig. 2b). To assess internalization by recipient cells, exosomes were labeled with the fluorescent dye PKH26 and co-cultured with MRC-5 embryonic lung fibroblasts for 6 h. Confocal microscopy revealed successful uptake and fusion of serum exosomes with MRC-5 cells (Fig. 2d).

To examine how serum exosomes indirectly affect thyroid carcinoma cells, MRC-5 fibroblasts were exposed to serum exosomes isolated from patients for 24 h. The culture supernatant was gathered and utilized for Cal62 thyroid cancer cell cultures (Fig. 2e). After 24 h of indirect co-culture, immunofluorescence staining revealed elevated the abundance of the proliferation-associated marker Ki67 in Cal62 cells compared to controls (Fig. 2f). EdU incorporation assays further confirmed enhanced proliferative activity (Fig. 2g). In addition, transwell migration assays demonstrated a pronounced enhancement of in Cal62 cell migration following treatment with the conditioned medium (Fig. 2h). These findings indicate that miRNAs delivered by serum exosomes from patients with lung metastatic thyroid cancer enhance the growth and motility potentials of thyroid carcinoma cells, likely through modulation of the tumor-supportive microenvironment mediated by MRC-5 fibroblasts.

### 3.3 Serum Exosomes Promote Thyroid Cancer Metastasis by Altering the Pulmonary Microenvironment

**Figure 3:**
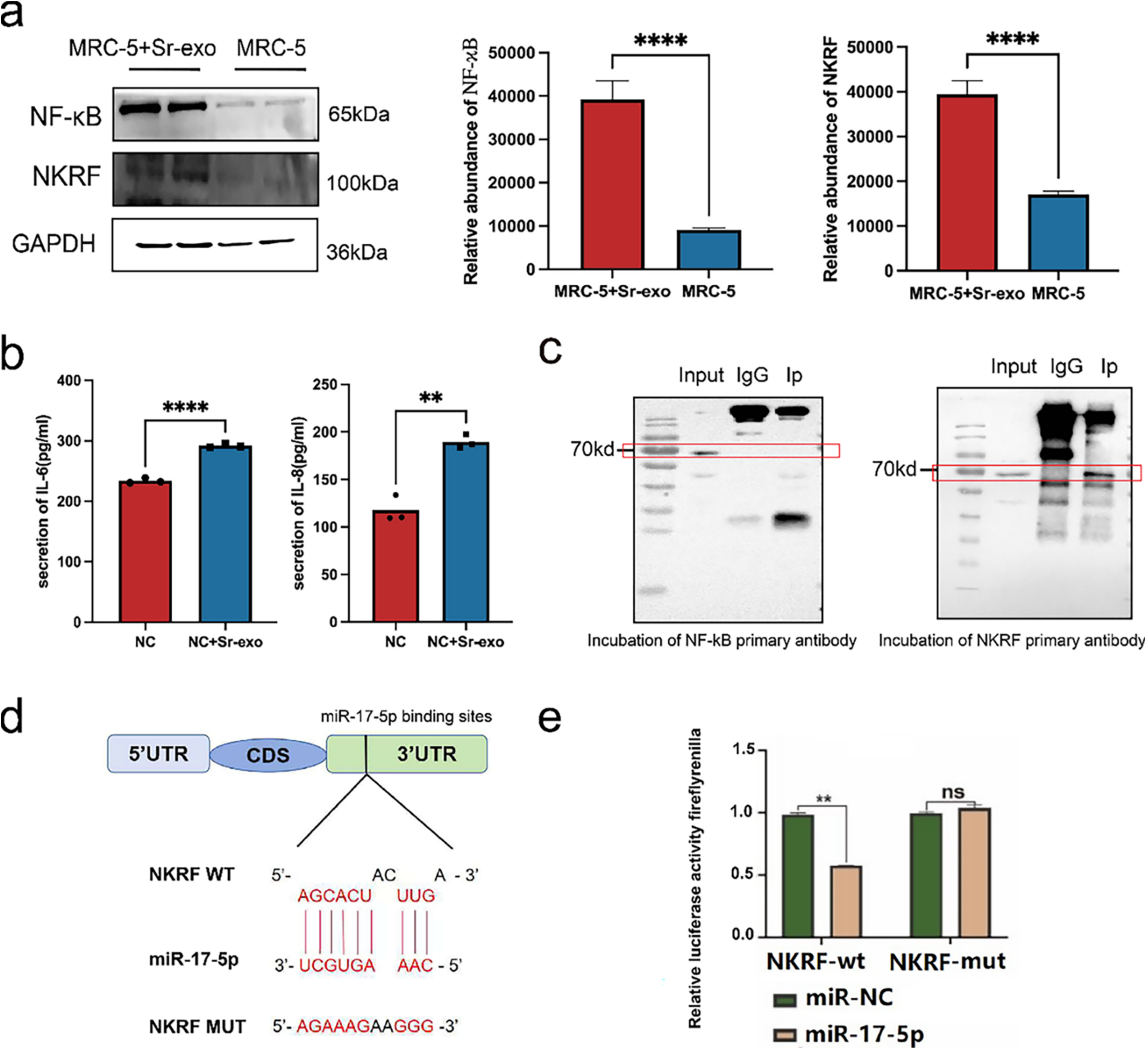
Serum exosomal miRNAs promote thyroid cancer lung metastasis by altering the pulmonary microenvironment. (**a**) WB analysis of NF-κB and NKRF protein levels in MRC-5 embryonic lung cells after treatment with serum-derived exosomes (Sr-exo) (n = 3). (**b**) Enzyme-linked immunosorbent assays (ELISA) results showing increased secretion of IL-6 and IL-8 from MRC-5 cells treated with Sr-exo compared to control (n = 3). (**c**) Co-immunoprecipitation (Co-IP) assay confirming the interaction between NF-κB and NKRF proteins in MRC-5 cells. (**d**) NKRF mRNA 3^′^-UTR (NKRF WT) binding site to miR-17-5p and designed NKRF 3^′^-UTR mutant (NKRF Mut) sequence. (**e**) Evaluation of the miR-17-5p–NKRF 3^′^-UTR interaction through dual-luciferase reporter assays (n = 3). ****p < 0.0001; **p < 0.01; ns, not significant.

Although miRNAs carried by serum-derived exosomes are involved in promoting lung metastasis of thyroid cancer, the specific mechanisms remain largely unclear. Previous studies have demonstrated that miRNAs can promote tumor progression by modulating the inflammatory microenvironment surrounding tumors [26]. Based on this, we hypothesized that exosomal miRNAs may influence the inflammatory status of embryonic lung fibroblasts, thereby facilitating lung metastasis in thyroid cancer. To explore this hypothesis, serum exosomes were isolated and co-cultured with MRC-5. After 24 h, WB analysis revealed increased expression of NF-κB and NKRF in the exosome-treated group compared to controls (Fig. 3a). ELISA further showed elevated levels of IL-6 and IL-8 in MRC-5 cells treated with serum exosomes (Fig. 3b).

To verify whether NKRF is directly targeted by miR-17-5p, Co-IP assays were conducted and confirmed a physical interaction between NF-κB and NKRF (Fig. 3c). Results from the dual-luciferase reporter analysis indicated that co-delivery of miR-17-5p and the NKRF WT significantly reduced luciferase activity, whereas no significant changes were observed with the NKRF Mut construct, indicating that NKRF is a direct target negatively regulated by miR-17-5p (Fig. 3d,e). These findings indicate that exosomal miRNAs activate inflammatory signaling in embryonic lung fibroblasts, reshaping the local microenvironment into a pro-inflammatory niche that promotes the colonization and the proliferation of thyroid cancer cells in pulmonary tissue.

### 3.4 miRNA Carried by Exosomes Targets NKRF in Embryonic Lung Cells

**Figure 4:**
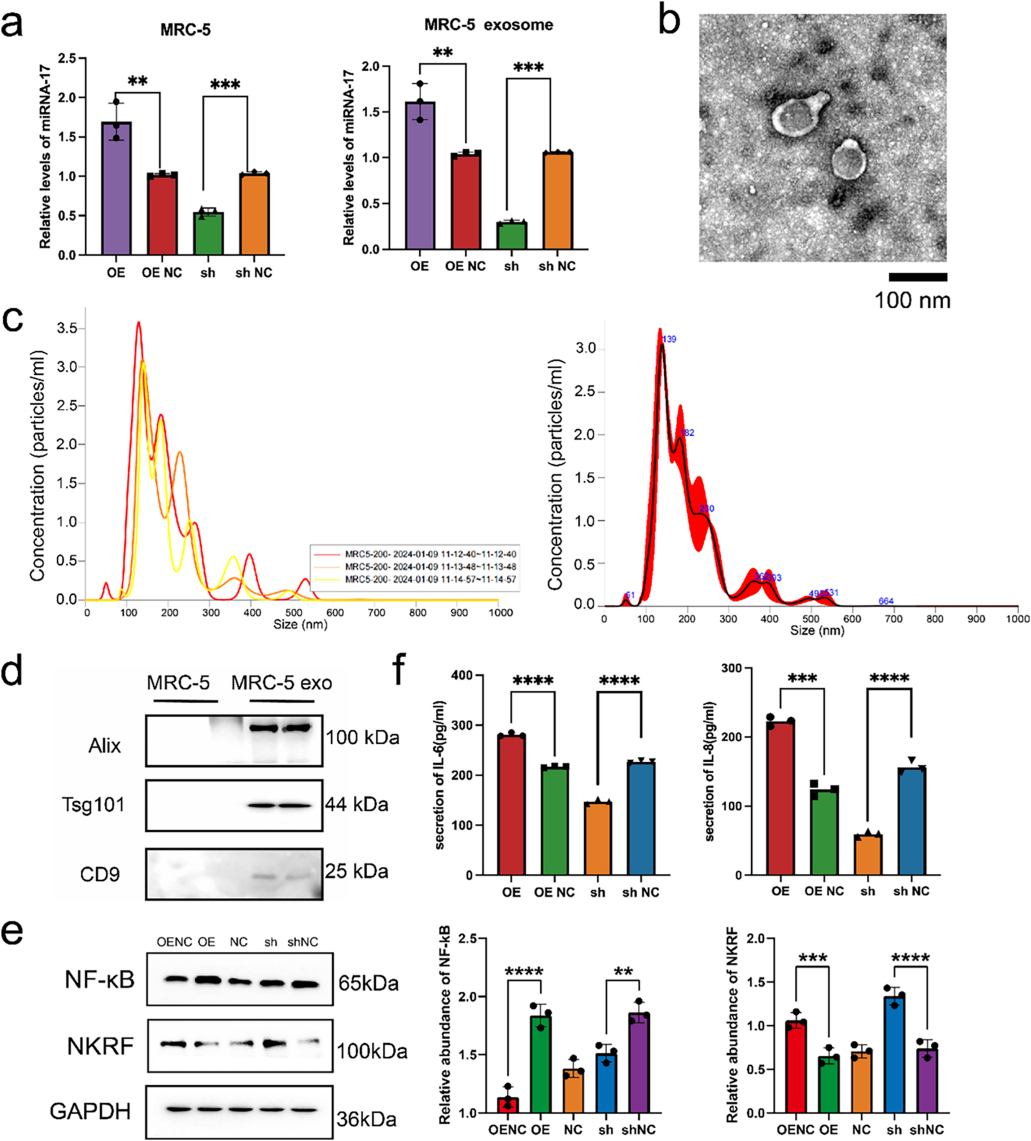
Exosomal miR-17-5p targets NKRF and activates inflammatory signaling in embryonic lung cells. (**a**) Relative expression levels of miR-17-5p in MRC-5 cells and their secreted exosomes following lentiviral overexpression (OE) or knockdown (sh) of miR-17-5p, as assessed by RNA isolation and quantitative (RT-PCR) (n = 3). (**b**) TEM visualization of exosomes purified from MRC-5 cell culture supernatants. (**c**) NTA showing the size distribution and concentration of MRC-5-derived exosomes. (**d**) WB analysis confirming the presence of canonical exosomal markers in purified exosome preparations. (**e**) WB analysis of NF-κB and NKRF protein levels in MRC-5 cells following miR-17-5p OE or sh (n = 3). (**f**) ELISA analysis showing the levels of IL-6 and IL-8 in MRC-5 cell supernatants after miR-17-5p modulation (n = 3). ****p < 0.0001; ***p < 0.001; **p < 0.01

Earlier findings have established miR-17 as a tumor-promoting factor in thyroid cancer. However, its specific targets in embryonic lung fibroblasts remain unclear. Previous studies have indicated that microRNAs can influence cellular behavior by interacting with cell surface receptors or modulating intracellular signaling pathways [19,26–28]. To identify potential downstream effectors of miR-17-5p in MRC-5 cells, lentiviral vectors were used to achieve miR-17-5p overexpression and silencing. RT-PCR confirmed effective modulation of miR-17 expression. Exosomes were subsequently isolated from culture supernatants via ultracentrifugation, and elevated miR-17 levels in exosomes were validated by RT-PCR (Fig. 4a). Transmission electron microscopy revealed that the isolated exosomes displayed the characteristic cup-shaped morphology (Fig. 4b). NTA revealed that the majority of particles ranged from 60 to 200 nm in diameter, with a predominant peak at approximately 139 nm. The particle concentration at this peak reached approximately 3.5 × 10^6^ particles/mL (Fig. 4c). WB analysis further confirmed the presence of canonical exosomal markers (Fig. 4d).

WB results revealed that overexpression of miR-17-5p increased NF-κB and decreased NKRF levels, while knockdown had the opposite effect (Fig. 4e). ELISA confirmed that IL-6 and IL-8 secretion was elevated in the overexpression group and reduced in the knockdown group (Fig. 4f). ELISA further demonstrated elevated IL-6 and IL-8 in the supernatants of miR-17-5p–overexpressing cells (Fig. 4f).

**Figure 5:**
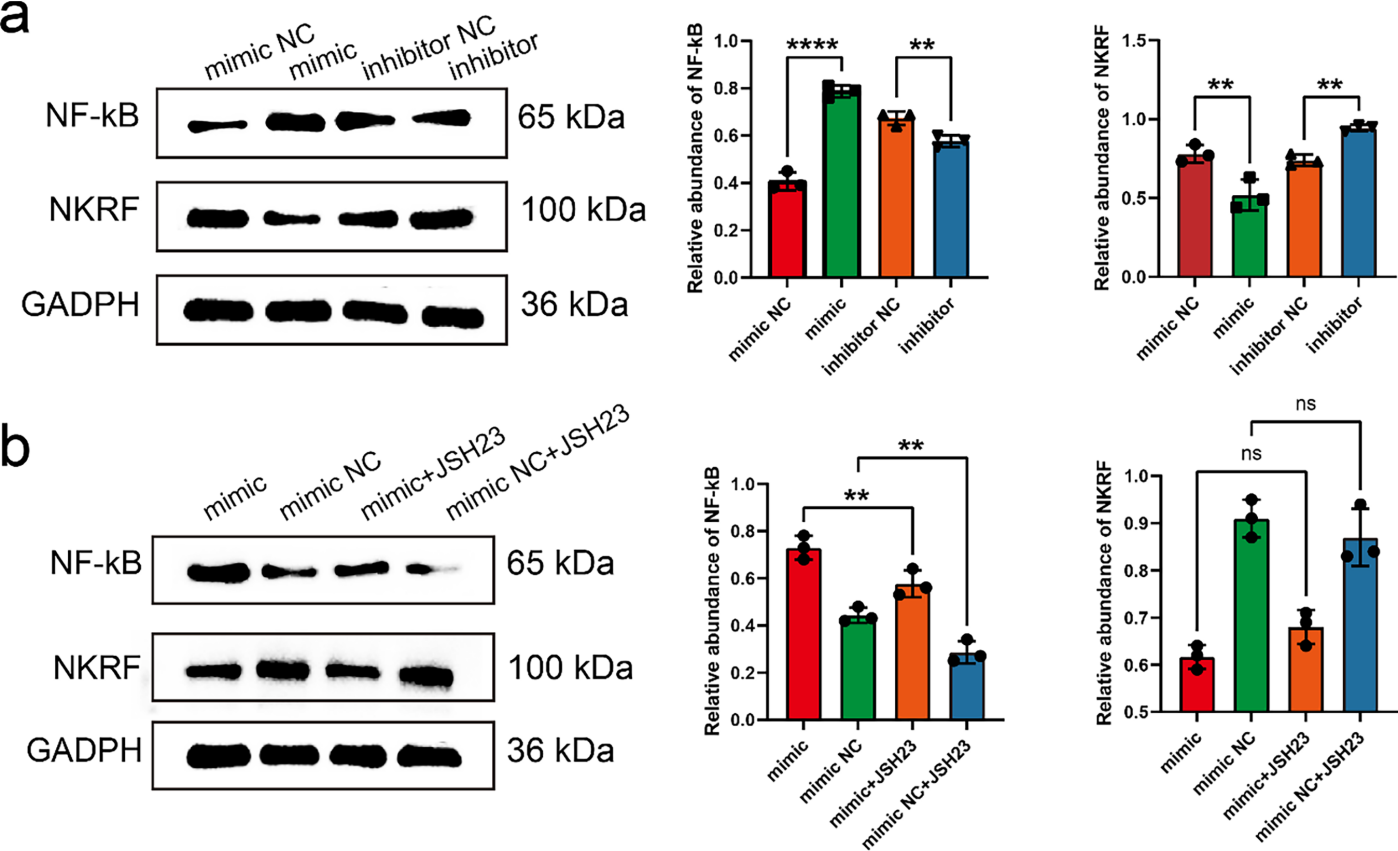
miR-17-5p modulates NF-κB signaling by targeting NKRF, and JSH-23 reverses its downstream effects. (**a**) WB analysis of NF-κB and NKRF protein levels in MRC-5 cells transfected with miR-17-5p mimic or inhibitor (n = 3), (**b**) and treated with the NF-κB inhibitor JSH-23 (n = 3). **p < 0.01; ****p < 0.0001; ns, not significant

To further elucidate the regulatory role of miR-17-5p in the NF-κB signaling pathway, WB analysis was performed to assess NF-κB and NKRF expression in MRC-5 cells transfected with miR-17-5p mimics or inhibitors. WB analysis revealed that miR-17-5p overexpression elevated NF-κB levels and reduced NKRF expression, while inhibition of miR-17-5p reversed these effects, indicating its role in activating NF-κB via NKRF suppression (Fig. 5a). Furthermore, NF-κB activation induced by miR-17-5p mimic was attenuated by JSH-23 treatment, whereas NKRF expression remained unaffected, confirming the involvement of NF-κB in this regulatory axis (Fig. 5b).

miR-17-5p delivered by exosomes suppresses NKRF in lung fibroblasts, enhancing inflammatory cytokine release and microenvironment formation. This mechanism may underlie the facilitation of thyroid cancer cell colonization and metastatic progression, warranting further investigation.

### 3.5 Exosome-miR-17 Targeting Embryonic Lung Cells Affects the Ability of Thyroid Cancer Cells

**Figure 6:**
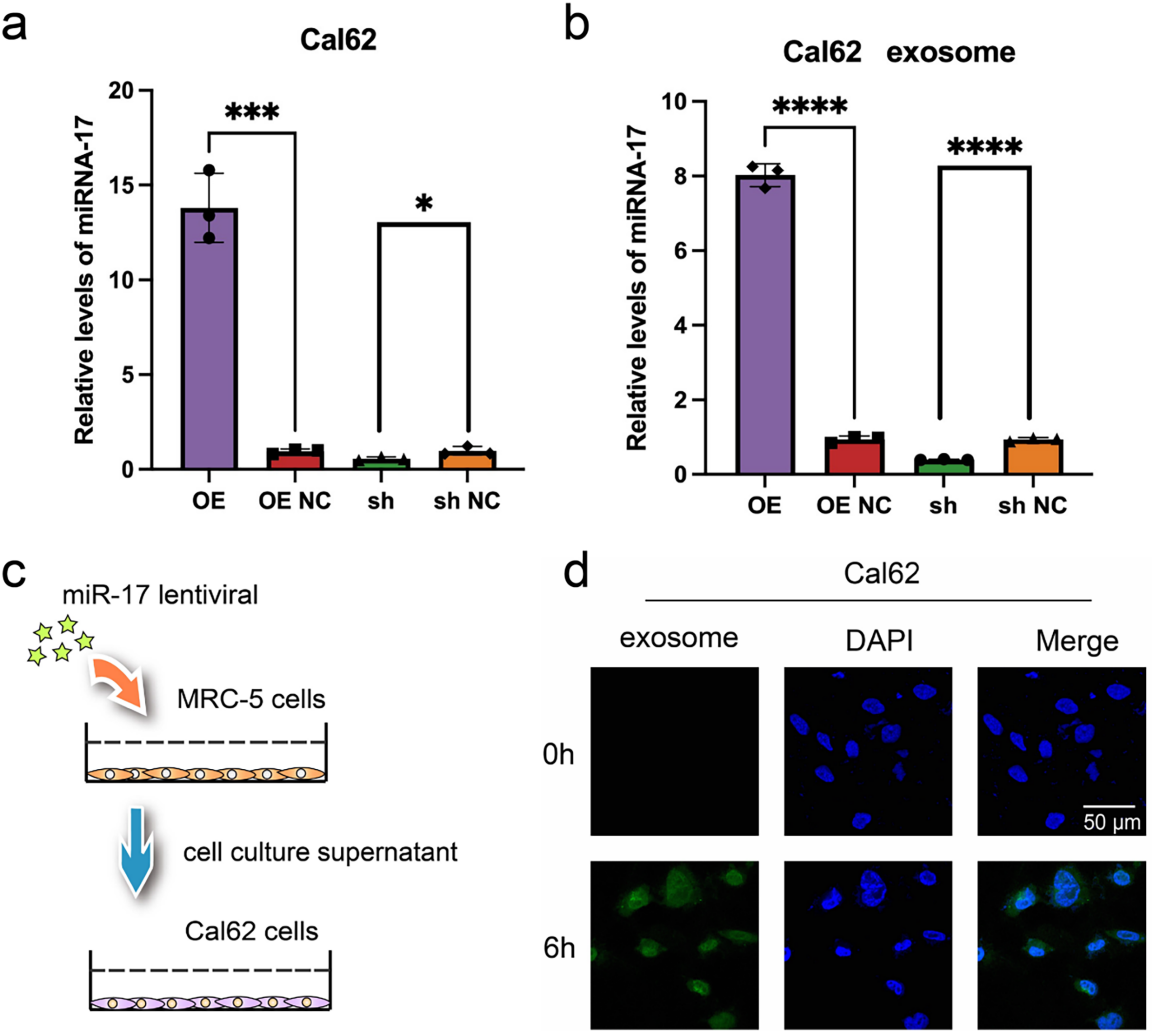
Validation of miR-17 and exosome uptake in Cal62 cells. (**a**,**b**) Detection of miR-17 expression in Cal62 cells **a** and their supernatant exosomes **b** after lentivirus transfection by RT-PCR assay (n = 3). (**c**) Schematic diagram of cell co-culture after lentivirus transfection. (**d**) Exosome tracer assay to verify the fusion of supernatant exosomes from MRC-5 cells with Cal62 cells. ****p < 0.0001; ***p < 0.001; *p < 0.05

Although exosomal miR-17 has been shown to target NKRF in embryonic lung fibroblasts and modulate the inflammatory microenvironment, its direct effect on thyroid cancer cells remains unclear. To address this, Cal62 thyroid cancer cells were transfected with lentiviral vectors to overexpress miR-17. RT-PCR confirmed successful upregulation of miR-17 in the transfected Cal62 cells (Fig. 6a), and elevated levels of exosomal miR-17 in the culture supernatants were also validated (Fig. 6b). To examine exosome-mediated communication, exosomes were obtained from culture media harvested from MRC-5 cells following transfection, labeled with the fluorescent dye PKH26, and co-incubated with wild-type Cal62 cells. Confocal microscopy confirmed efficient uptake of exosomes by Cal62 cells after six hours of co-culture (Fig. 6c,d).

**Figure 7:**
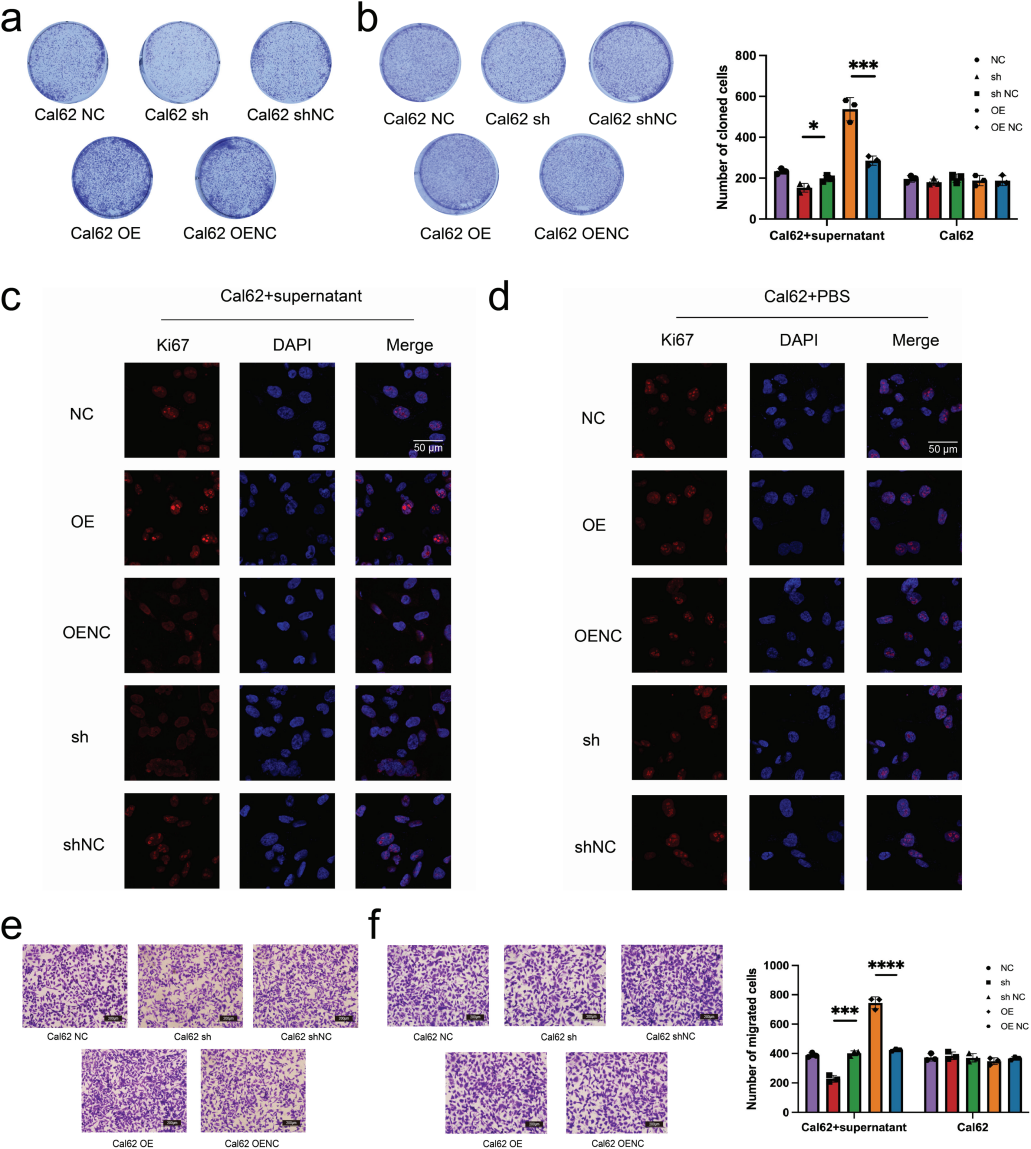
Exosomal miR-17 enhances Cal62 proliferation and migration more effectively than direct overexpression. (**a**,**b**) Plate cloning assays were performed to test the proliferative capacity of wild Cal62 co-cultured with MRC-5 cell supernatants after transfection with miR-17 as well as Cal62 cells transfected with miR-17 alone (n = 3). (**c**,**d**) Immunofluorescence assay to detect Ki67 content in Cal62 cells. (**e**,**f**) The cell migration assay detects the migration ability of Cal62 cells (n = 3). ****p < 0.0001; ***p < 0.001; *p < 0.05.

Colony formation assays revealed a marked increase in the proliferative capacity of Cal62 cells co-cultured with transfected MRC-5 supernatants, compared to those directly transfected with miR-17 (Fig. 7a,b). Ki67 expression, a marker of cell proliferation, was further evaluated by immunofluorescence. Significantly higher Ki67 levels were identified in Cal62 cells co-cultured with supernatants from miR-17–transfected MRC-5 cells compared to those directly transfected with miR-17 lentivirus (Fig. 7c,d). In addition, cell migration assays demonstrated that co-cultured Cal62 cells displayed enhanced migratory capacity compared to directly transfected cells (Fig. 7e,f). Overall, these data reveal that exosomal miR-17 influences embryonic lung fibroblasts and promotes thyroid cancer cell proliferation and migration through shaping a supportive microenvironment.

### 3.6 In Vivo Validation of miR-17-5p in Thyroid Cancer Growth and Lung Metastasis

**Figure 8:**
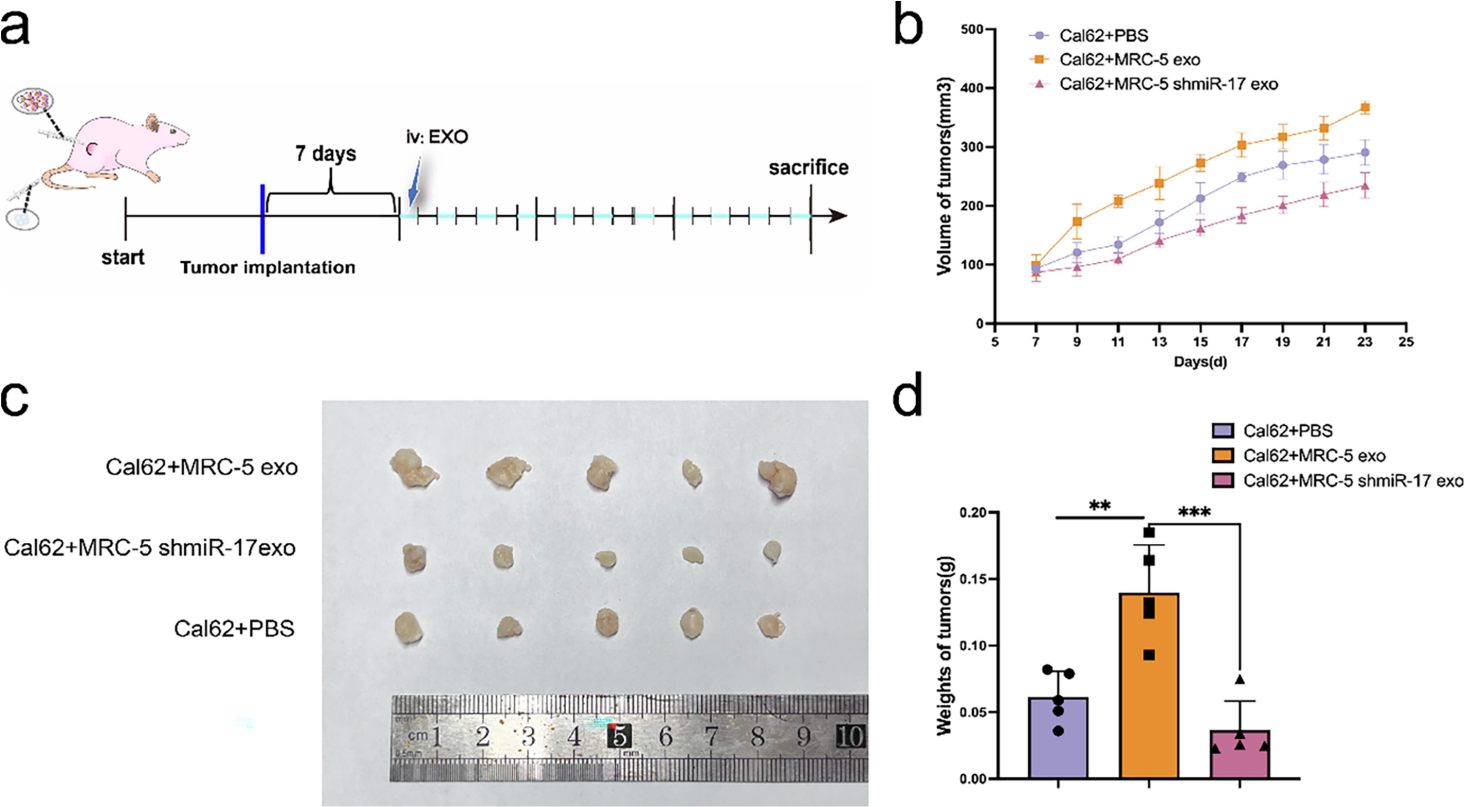
Exosomal miR-17-5p accelerates subcutaneous thyroid cancer growth *in vivo*. (**a**) Schematic diagram of subcutaneous implantation and exosome injection in animal experiments. (**b**) Tumor volume was measured at regular intervals (n = 5). (**c**) Representative images of tumors collected from each group at the endpoint (n = 5). (**d**) Tumor weights were measured and statistically analyzed (n = 5). ***p < 0.001; **p < 0.01

To evaluate the *in vivo* effects of miR-17-5p, a subcutaneous xenograft model was initially generated in nude mice using Cal62 thyroid cancer cells. Following tumor formation, exosomes derived from miR-17-5p–overexpressing MRC-5 cells received tail-vein injections every other day, while control mice received PBS (Fig. 8a). Tumor volume and mass were significantly was elevated in the miR-17-5p group relative to the controls (Fig. 8c,d), indicating that exosomal miR-17-5p facilitates thyroid cancer cell growth *in vivo*.

**Figure 9:**
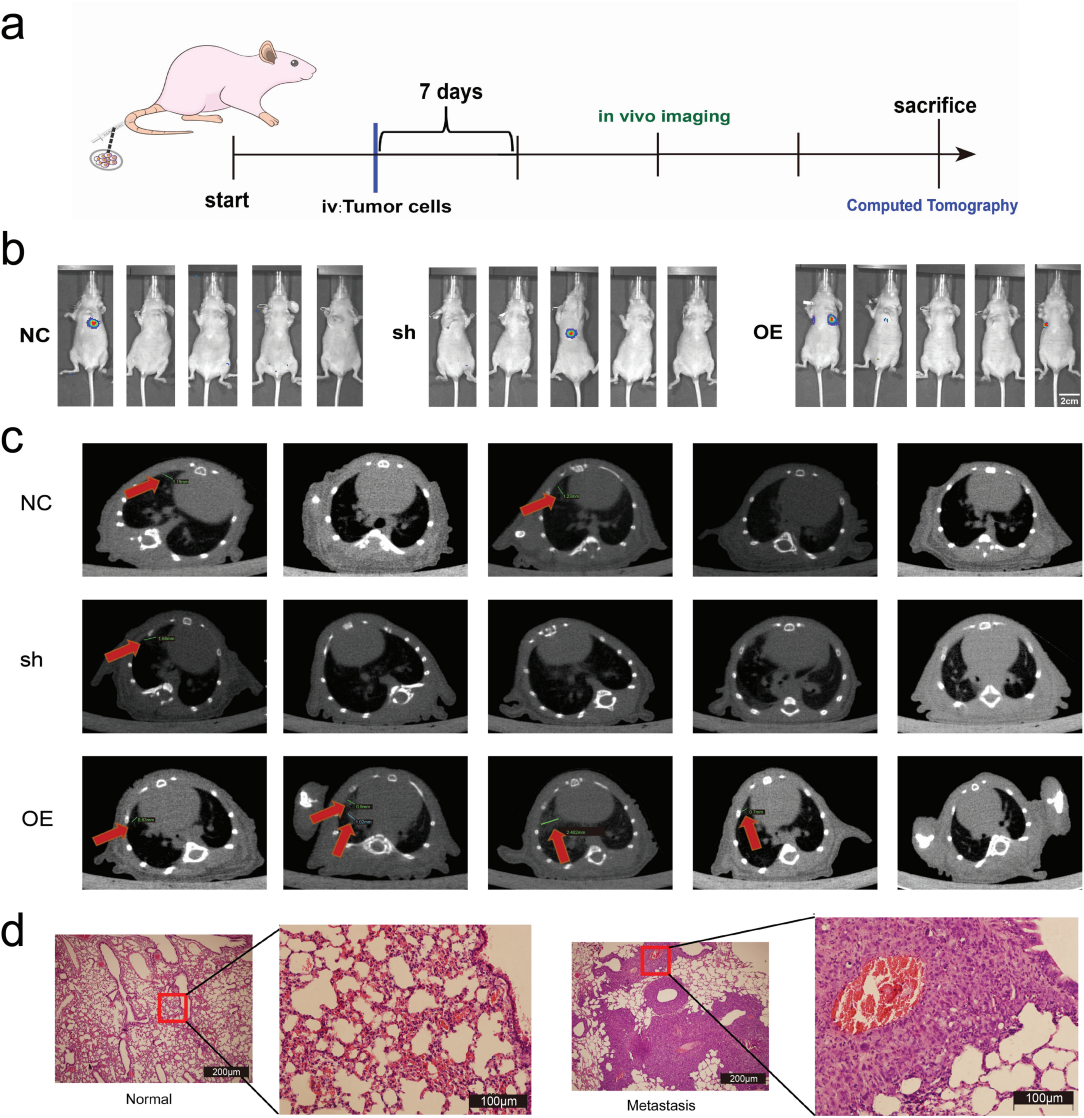
Exosomal miR-17-5p enhances lung metastasis of thyroid cancer *in vivo*. (a) Schematic diagram of the lung metastasis model of thyroid cancer in animal experiments. (**b**) *In vivo* imaging of lung metastases in mice (n = 5). (**c**) Computed tomography images of lung metastases (indicated by red arrows) in mice (n = 5). (**d**) Comparison of HE staining of lungs of mice with lung metastases with normal mice

To evaluate the metastatic role of miR-17-5p, a lung metastasis model was generated by tail-vein injection of Cal62 cells overexpressing miR-17-5p (Fig. 9a). *In vivo* imaging at week 4 and micro-CT at week 5 revealed enhanced lung metastasis in the miR-17-5p group (Fig. 9b,c). H&E staining verified metastatic nodule formation in lung tissues (Fig. 9d). Our results show that miR-17-5p promotes both growth and pulmonary metastasis of thyroid cancer cells *in vivo*.

**Figure 10:**
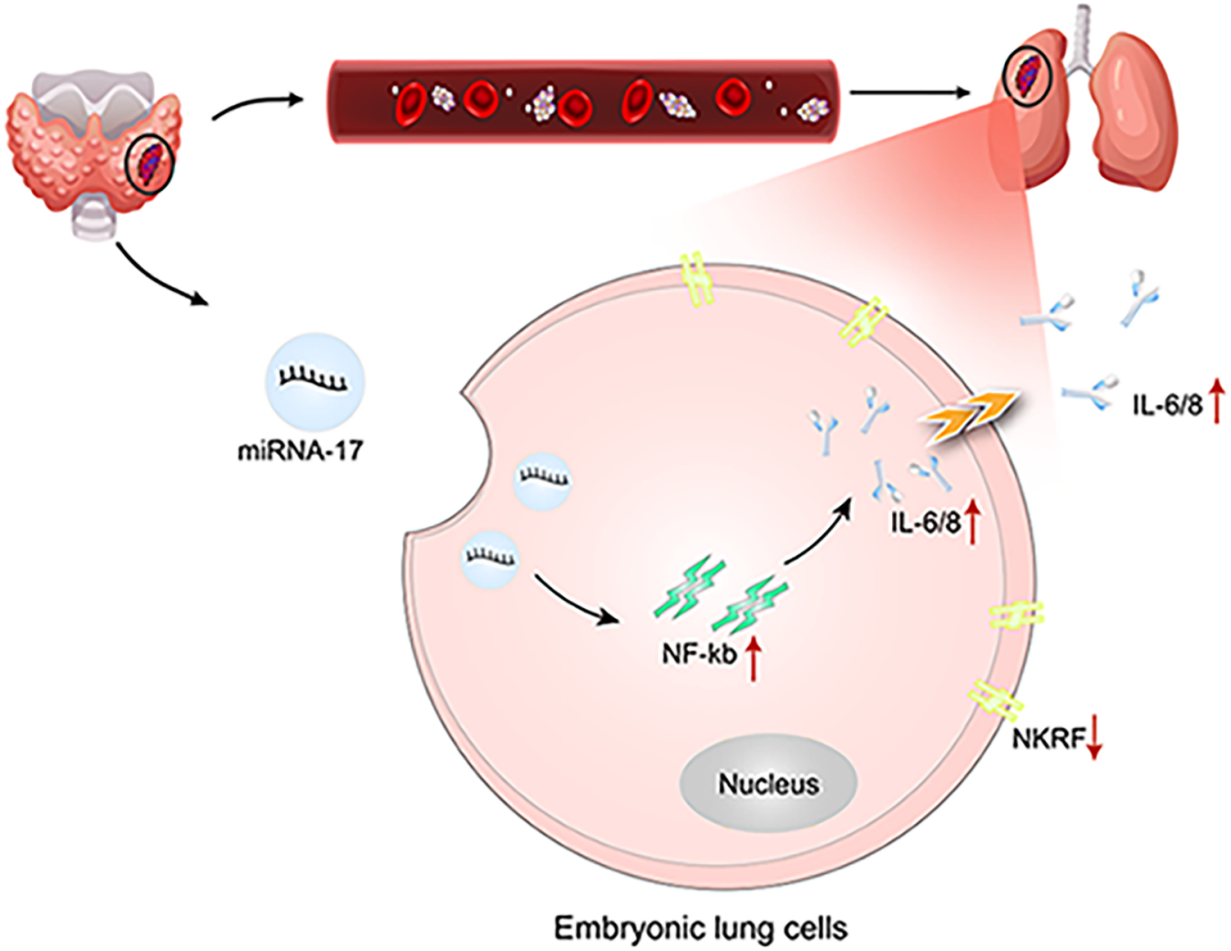
Schematic diagram of the study (created using Adobe Illustrator). Red upward arrows indicate increased expression or activation, whereas downward arrows indicate reduced expression

Our study demonstrates that, during the progression of thyroid cancer, exosomes enriched with miR-17 are secreted by the primary tumor and enter the circulatory system. These exosomes target embryonic lung cells, where they induce NF-κB signaling and enhance the release of IL-6 and IL-8. This creates a pro-inflammatory microenvironment that serves as a fertile “soil” for the colonization and metastasis of thyroid cancer cells in the lungs (Fig. 10).

## 4 Discussion

The “seed and soil” concept is a well-established paradigm in tumor biology, offering a theoretical basis for explaining why certain cancers preferentially metastasize to specific organs, such as in gastric and other malignancies [11,29,30]. However, its significance in thyroid cancer has not been thoroughly defined. Clinically, undifferentiated thyroid cancer tends to spread most frequently to the lungs, and such metastases are strongly linked to worse survival outcomes [2].

To explore the molecular drivers of this lung tropism, we conducted miRNA profiling on serum-derived exosomes collected preoperatively from patients with pulmonary metastases. Several miRNAs were significantly more abundant in the metastatic group, with miR-17 showing the greatest increase. As part of the oncogenic miR-17-92 cluster, miR-17 has been documented to be overexpressed in a variety of cancers, including B-cell lymphoma, lung cancer, colorectal carcinoma, and hepatocellular carcinoma [31–33]. This miRNA cluster is known to influence tumor biology through multiple pathways, such as stimulating cell proliferation, blocking apoptosis, promoting angiogenesis, triggering inflammation, and facilitating metastatic spread [32,33]. In this study, we demonstrated that exosomal miR-17 derived from thyroid cancer cells activated NF-κB signaling in pulmonary fibroblasts. This activation enhanced IL-6 and IL-8 expression, fostering a pro-inflammatory microenvironment that promoted lung metastasis *in vivo*.

From a clinical application perspective, the higher serum exosomal miR-17 levels observed in patients with lung metastases suggest its application as a non-invasive diagnostic tool for early-stage assessment and risk prediction. Furthermore, due to its role in regulating inflammatory pathways, miR-17 may constitute a potential therapeutic target. Approaches such as antisense oligonucleotides (ASOs) or locked nucleic acid (LNA) inhibitors could be explored to block pre-metastatic niche formation and slow metastatic progression [34]. Collectively, our results offer an initial mechanistic insight into the contribution of exosomal miR-17 to lung metastasis in thyroid cancer and open avenues for potential translational applications.

Several limitations should be noted. The number of patients enrolled was relatively small, and confirmation in larger, independent cohorts is required. The *in vitro* experiments were conducted with embryonic lung fibroblasts, which may not fully represent the biological features of adult lung stromal cells. Although the data support a role for miR-17 in promoting metastasis, the downstream pathways and their interplay with other factors within the metastatic niche are not yet fully understood. Exosomes were identified using TEM, NTA, and specific protein markers. However, ultracentrifugation may not completely remove other extracellular vesicles such as microvesicles, which could influence the observed outcomes. Future research should incorporate more precise vesicle purification methods and employ physiologically relevant models, such as lung organoids, to validate and expand these findings.

## Data Availability

The data that support the findings of this study are available from the corresponding author, Junyi Wang, upon reasonable request.
